# Impact of γ-rays Irradiation on Hybrid TiO_2_-SiO_2_ Sol-Gel Films Doped with RHODAMINE 6G

**DOI:** 10.3390/ma14195754

**Published:** 2021-10-02

**Authors:** Maxime Royon, Francis Vocanson, Damien Jamon, François Royer, Emmanuel Marin, Adriana Morana, Cosimo Campanella, Aziz Boukenter, Youcef Ouerdane, Yves Jourlin, Sylvain Girard

**Affiliations:** Laboratoire H. Curien, UJM-CNRS-IOGS, Université de Saint-Etienne, 18 rue du Pr. Benoît Lauras, 42000 Saint-Etienne, France; francis.vocanson@univ-st-etienne.fr (F.V.); damien.jamon@univ-st-etienne.fr (D.J.); francois.royer@univ-st-etienne.fr (F.R.); emmanuel.marin@univ-st-etienne.fr (E.M.); adriana.morana@univ-st-etienne.fr (A.M.); cosimo.campanella@univ-st-etienne.fr (C.C.); aziz.boukenter@univ-st-etienne.fr (A.B.); ouerdane@univ-st-etienne.fr (Y.O.); yves.jourlin@univ-st-etienne.fr (Y.J.); sylvain.girard@univ-st-etienne.fr (S.G.)

**Keywords:** Rhodamine 6G, sol-gel films, photoluminescence, γ-rays, FTIR spectroscopy, photobleaching

## Abstract

In the present paper, we investigate how the optical and structural properties, in particular the observed photoluminescence (PL) of photocurable and organic-inorganic TiO_2_-SiO_2_ sol-gel films doped with Rhodamine 6G (R6G) are affected by γ-rays. For this, four luminescent films, firstly polymerized with UV photons (365 nm), were submitted to different accumulated doses of 50 kGy, 200 kGy, 500 kGy and 1 MGy while one sample was kept as a reference and unirradiated. The PL, recorded under excitations at 365 nm, 442 nm and 488 nm clearly evidences that a strong signal peaking at 564 nm is still largely present in the γ-irradiated samples. In addition, M-lines and Fourier-transform infrared (FTIR) spectroscopies are used to quantify the radiation induced refractive index variation and the chemical changes, respectively. Results show that a refractive index decrease of 7 × 10^−3^ at 633 nm is achieved at a 1 MGy accumulated dose while a photo-induced polymerization occurs, related to the consumption of CH=C, Si-OH and Si-O-CH_3_ groups to form Ti-O and Si-O bonds. All these results confirm that the host matrix (TiO_2_-SiO_2_) and R6G fluorophores successfully withstand the hard γ-ray exposure, opening the way to the use of this material for sensing applications in radiation-rich environments.

## 1. Introduction

The sol-gel process is a powerful technique used for the manufacturing of low-cost transparent materials. Based on hydrolysis and condensation reactions [1], this chemistry route clearly evidences its impact by the diversity and abundance of applicative fields. Among all these applications, we can mention the development of protective coatings to prevent corrosion phenomena [2,3], sol-gel layers possessing flame-retardant properties [4], gas and pH sensors [5,6]. The sol-gel is also widely used in the field of photonics, such as the fabrication of optical waveguides with low losses operating in the telecommunication ranges [7,8] where applications can be found in the aerospace domain [9] or diffraction gratings [10,11] for producing antireflection layers in the solar energy field [12]. In our case, the potential of the sol-gel material is investigated in the framework of the ADD-ON European project targeting to develop new generation of sol-gel based sensors for structural health monitoring of aircrafts [9].

More specifically, regarding the sol-gel elaboration, metal alkoxides precursors are exploited in order to generate an inorganic metal oxides matrix such as TiO_2_, ZrO_2_, SiO_2_ or combinations thereof. In addition, a significant advantage relies in tailoring the produced material by varying the precursor concentration thus adjusting their optical or mechanical properties. Using precursors containing an organic chain is of great interest since a UV photosensitivity can be induced while it can also be achieved (or enhanced) by incorporation of commercial photoinitiators [13,14]. This opens the way to the creation of photopatternable materials by coupling amplitude photomask lithography or direct laser writing techniques [9,15]. Basically, photosensitive sol-gel layers are known to act as negative photoresists: the region insolated through UV photons is polymerized implying a densified pattern. After development in a proper alcoholic solution, the unexposed zone is dissolved revealing the final structure. Interestingly, additional functionalities can be obtained by incorporating specific molecules or nanocrystals during the sol-gel elaboration. It is thus possible to have access to photocurable sol-gel dedicated to magneto-optic application by adding cobalt ferrite [16] or even to the creation of fluorescent materials [17]. Among all the proposed fluorophores, Rhodamine 6G (R6G) has attracted particular attention for several years and was largely studied [18,19,20,21]. This organic dye, from the xanthene family, is extensively used in optics, spectroscopy and in laser fields due to its intense fluorescence namely in the 500–600 nm range [22]. Moreover, several applications can be found in the literature where the evolution of its luminescent properties can be of interest regarding potential temperature sensing abilities [23] or in more exotic fields such as biomarkers [24]. R6G is known to be degraded after intense light exposure inducing a decrease in the fluorescence efficiency [18] namely after probing in the ultraviolet (UV) region [25]. To our knowledge, no study was performed regarding the impact of γ-ray irradiation on such dye. In this paper, a detailed investigation on the PL properties of a hybrid TiO_2_-SiO_2_ sol-gel doped with R6G is presented. After being γ-irradiated with several accumulated doses up to 1 MGy (1 Gy = 1 J/kg), the samples were characterized by PL, Fourier-transform infrared (FTIR) and M-lines spectroscopies in order to check a potential impact of hard γ-rays exposure. Our objective is to assess if the properties of those materials, particularly their luminescence responses, could be degraded by the X-ray imaging inspections of the aircrafts during their maintenance phase.

## 2. Materials and Methods

**Figure 1:**
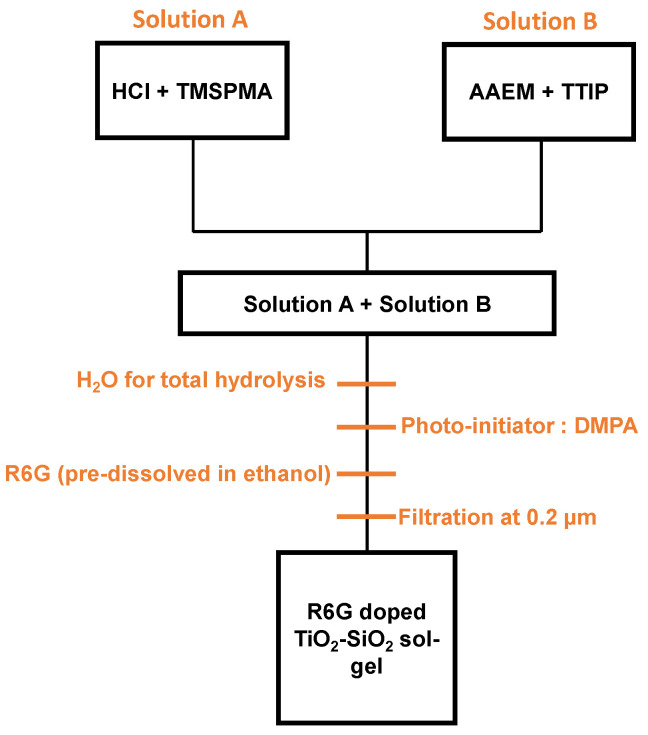
Elaboration of the TiO_2_-SiO_2_ sol-gel doped with Rhodamine 6G (R6G) in the form of 1wt.%. R6G is pre-dissolved in ethanol before incorporation in the solution. Adapted from [26].

For all the experiments presented in this manuscript, we have used an organic-inorganic TiO_2_-SiO_2_ sol-gel whose chemical reagents were provided by Sigma-Aldrich (Saint-Quentin Fallavier, France). This latter was already investigated and developed in our previous studies [9,26] and a more detailed description is given in Figure 1. The proposed sol-gel is composed by titanium (IV) isopropoxide (TTIP) and 3-(trimethoxysilyl)propyl methacrylate (TMSPMA) acting as titanium and silicate precursors respectively. In particular, two preliminary solutions are prepared: TMSPMA is mixed with HCl so as to achieve a partial hydrolysis (solution A) while TTIP is chelated with 2-(methacryloyloxy)ethyl acetoacetate (AAEM) and denoted as solution B. The corresponding molar ratios Ti:Si:AAEM are 10:10:5.5. Solutions A and B are mixed and distilled water is incorporated to obtain a total hydrolysis. As mentioned earlier, using an organic-inorganic sol-gel allows having access to a UV photosensitivity. Although our sol-gels are photosensitive with respect to UV photons or X-rays [26], a 2,2-dimethoxy-2-phenylacetophenone (DMPA) photo-initiator in the form of 0.7 wt.% is added to enhance the photopolymerization. In order to have access to fluorescent sol-gels, we have incorporated Rhodamine 6G (dye content 95%), an organic compound widely used in laser due to the very high quantum yield of fluorescence and emission in the visible part between roughly 500 and 600 nm [22]. To this aim, R6G is pre-dissolved in ethanol and added to the mixture in the form of 1wt.%. The choice of ethanol is made among several organic solvents regarding a previous study where authors showed that a high solubility is achieved without any significant influence in the fluorescence efficiency [27]. After the solution elaboration, the mixture is filtered at 0.2 µm and aged for 2 days before any use.

**Figure 2:**
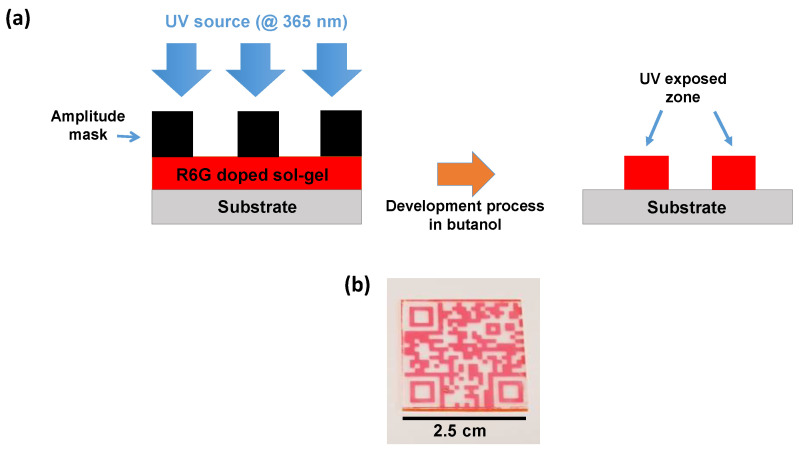
Example of photolithography process. (**a**) Photomask lithography technique on R6G TiO_2_-SiO_2_ sol-gel layers. (**b**) Picture of a pattern (QR-code) that can be achieved using the UV photomask approach. The red part corresponds to the UV exposed part and is still present after development in a butanol solution. All the doped layers are exposed to UV photons (365 nm) at 180 J/cm^2^.

The hybrid silica-titania sol-gels are then coated on several substrates (borofloat33, silicon or soda-lime glass) depending on the performed characterization. All the layers were deposited in clean room at fixed hygrometry and temperature using the spin coating approach, resulting in film thicknesses of 1.9 µm obtained by profilometer measurements. After the deposition, the sol-gel was pre-baked at 60 °C during 5 min to pre-densify the layer but also to evaporate the solvents. The photolithography process is a necessary step in order to induce the polymerization of the layers and by extension the creation of patterns. For this purpose, the insolation was performed using a UV chamber consisting on a Light Emitting Diode (LED) matrix providing UV photons at 365 nm (3.4 eV). This latter results in a 10 cm squared light source and the distance between the LEDs and the films can be adjusted so that the fluence can be easily controlled. All the samples were submitted to a 180 J/cm^2^ fluence. Interestingly, patterns can be created by coupling UV insolation and an amplitude photomask placed along the optical path above the substrate with the sol-gel layer as shown in Figure 2a. Insofar as the sol-gel based organo TiO_2_-SiO_2_ reacts as a negative photoresist, the regions that have not been exposed to UV photons are dissolved during the development process in a butyl alcohol solution revealing an example of pattern (QR-code) represented in Figure 2b. For this latter, the red zones correspond to the R6G doped TiO_2_-SiO_2_ sol-gel exposed to UV photons. Finally, after the light exposure and the development process, all the layers were submitted to a final thermal treatment at 90 °C during 30 min in order to stabilize the films.

In order to highlight a possible impact of hard γ-ray photons on the luminescent properties and on the degradation of the sol-gel layers, the different samples containing the UV exposed layers were submitted to a ^60^Co γ-ray source (1.17 and 1.33 MeV) at the IRSN IRMA facility (Saclay, France). The distance between the ^60^Co source and the layers governs the dose rate (Gy/s) thus defining the total accumulated dose. Four samples were then irradiated at different dose rates of 0.13 kGy/h, 0.52 kGy/h, 1.3 kGy/h and 2.6 kGy/h resulting in accumulated doses of 50 kGy, 200 kGy, 500 kGy and 1 MGy, respectively, while a pristine sample (only UV exposure) was kept as a reference (0 Gy).

The layers were characterized in terms of PL via a Horiba Jovin Yvon confocal microspectrometer (Longjumeau, France) using a He-Cd laser operating at 442 nm (2.81 eV) and an Argon ion (Ar^+^) laser providing photons at 488 nm (2.54 eV), where their respective powers can be adjusted using filters from 0.01% to 100% of the total laser power. For each configuration, the different sources are focused into the sol-gel layers. More specifically, an Olympus Mplan N100x (NA = 0.9) is used for the two different visible wavelengths (442 nm and 488 nm). All the PL spectra were recorded with a 150 l/mm grating, thus defining a spectral resolution of 0.376 nm. In addition to these two excitation wavelengths, a UV lamp (UVLS-28) providing a uniform and intense light source at 365 nm (8 W) is employed to visually check the impact of γ-rays on the PL of the structured sol-gel patterns. The different layers were also characterized using a Nicolet iS20 Fourier transform infrared (FTIR) spectrometer (Thermo Scientific, Waltham, MA, USA) operating in attenuated total reflectance (ATR) with a resolution of 0.482 cm^−1^. For this latter, the sol-gel layers should be deposited on Si substrates as it is transparent in the far infra-red (IR) region. A Cary series UV-Vis-NIR spectrophotometer (Agilent technologies, Santa Clara, CA, USA) with a 1 nm resolution is employed to have access to the impact of the R6G incorporated in the TiO_2_-SiO_2_ solution namely in terms of absorption bands. Finally, the refractive index at 633 nm is evaluated using the M-lines spectroscopy [28] at room temperature. This technique being based on the optical guided modes propagating inside the sol-gel layer, it is thus crucial to have the most important refractive index contrast between the layer and the substrate. To this particular investigation, the films were deposited on a borofloat 33 glass substrate, whose refractive index is 1.47 at 633 nm while the TiO_2_-SiO_2_ one is roughly 1.58 ensuring important guiding properties.

## 3. Results

### 3.1. Absorption and PL Characterization of R6G Doped Sol-Gel Films

**Figure 3:**
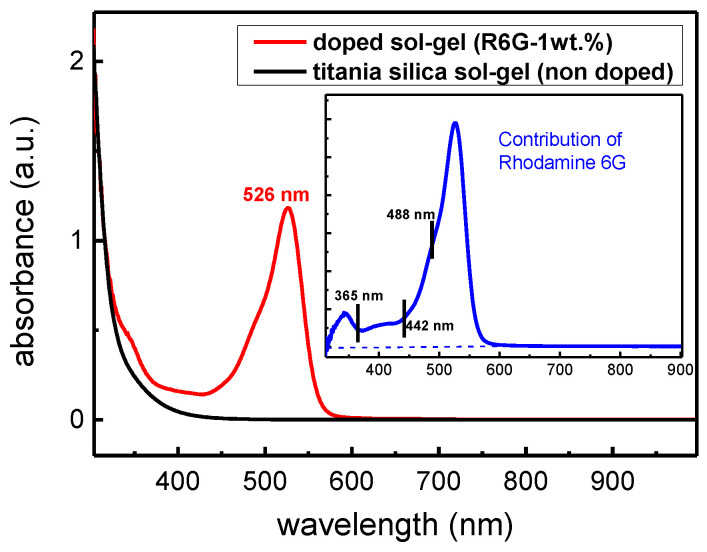
Absorption spectra obtained before γ irradiation on a titania-silica sol-gel film (black line) and a R6G doped sample at 1wt.% (red line). The inset shows the contribution of R6G and corresponds to the absorption difference between the doped and the undoped samples.

This part is dedicated to the impact of the R6G incorporation in the TiO_2_-SiO_2_ sol-gel. We underline that the results presented in this section correspond to pristine sample (0 Gy) thermally treated only and exposed to UV photons (180 J/cm^2^), as described in Section 2. To this aim, absorption spectroscopy in the [300 nm–1000 nm] spectral range was performed in order to have access to potential bands induced by the incorporation of R6G molecules in TiO_2_-SiO_2_ sol-gels. Figure 3 reveals spectra of films prepared from an undoped titania-silica sol-gel film (black line) and a R6G doped TiO_2_-SiO_2_ film at 1wt.% (red line). It is important to mention that the given spectra correspond to the sole response of the films as the soda-lime glass substrate was taken as reference when acquiring the absorbance signatures. First of all, the undoped film is transparent in the visible range while a strong absorption is present in the UV region, as already observed by other authors regarding titania-silica based materials with different chelating agents [29]. The absorption spectrum of the doped film highlights the effect of R6G incorporation with the presence of an intense absorption band peaking at 526 nm. More specifically, by making the absorption difference between the doped film and the undoped one, as shown in the inset of Figure 3, we can clearly reveal the contribution of R6G with additional absorptions ranging from roughly 330 nm to 570 nm. The three wavelengths (365 nm, 442 nm and 488 nm) used for probing the luminescence of the samples are also highlighted showing that weak and strong absorption regions are investigated.

**Figure 4:**
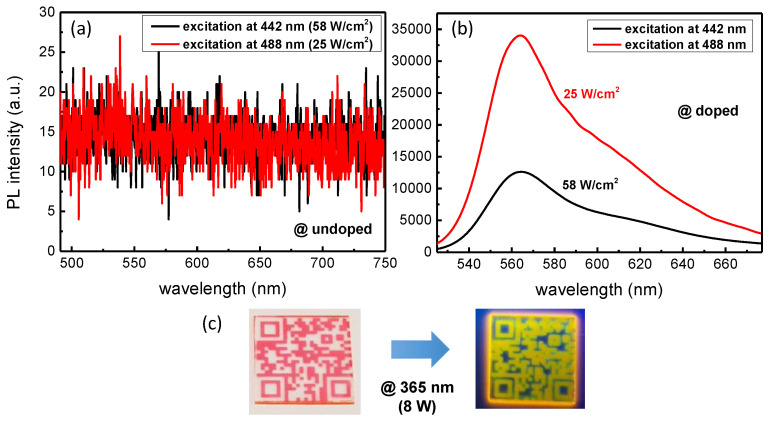
PL study under excitation at 442 nm and 488 nm for an undoped TiO_2_-SiO_2_ sol-gel film (**a**) and a R6G doped sol-gel layer (**b**). A visual observation of the PL is given under excitation of a QR-code architecture (25 × 25 mm^2^) at 365 nm (**c**).

In addition to the absorption, the layers were characterized in terms of PL. Figure 4a reveals the PL signal after probing an undoped sol-gel film using lasers operating at 442 nm (black line) and 488 nm (red line) with power densities of 58 W/cm^2^ and 25 W/cm^2^, respectively. Through this graph, it is obvious that no PL signal is present under excitation with these two laser sources. The R6G doped sol-gels were also probed and the results are synthesized in Figure 4b. A very intense PL can be achieved under excitation at 442 nm (black line) and 488 nm (red line). In both cases, we can observe an emission band peaking at roughly 564 nm. Despite a lower power density, we note that a stronger PL signal is obtained for a 488 nm excitation. This behavior was expected since a more important absorption occurs at this particular wavelength compared with 442 nm as illustrated in Figure 3. Finally, visual observations regarding the PL are presented in Figure 4c. On the left is imaged a QR-code architecture (25 × 25 mm^2^) under visible light while the same structure shows a strong luminescence under excitation at 365 nm (8 W). This clearly evidences a stokes shift from a green absorption (peaking at 526 nm) to a yellow-orange fluorescence (peaking at 564 nm) and a successful incorporation of R6G into the TiO_2_-SiO_2_ sol-gel matrix.

### 3.2. Photobleaching under Visible Light Exposure

**Figure 5:**
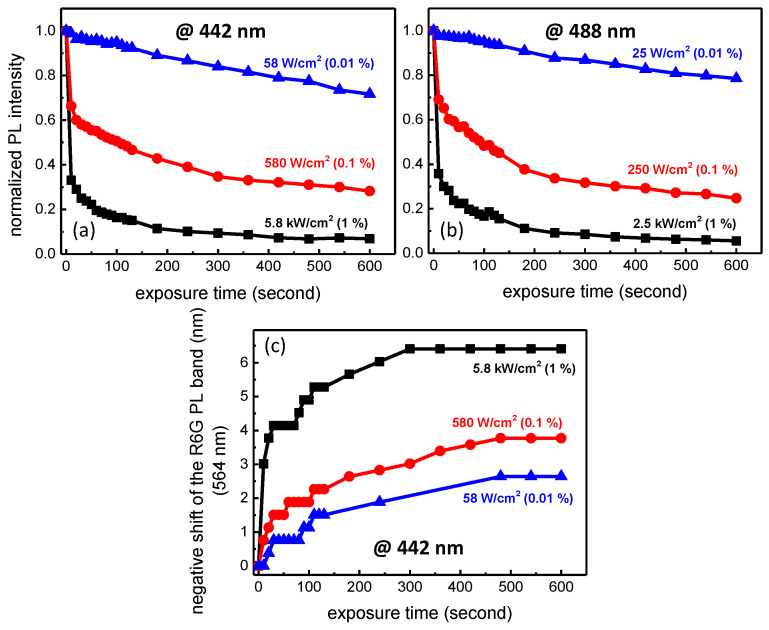
Degradation of the R6G luminescence under excitation at 442 nm with power densities of 5.8 kW/cm^2^, 580 W/cm^2^ and 58 W/cm^2^ (**a**). Degradation of the PL under excitation at 488 nm (2.5 kW/cm^2^, 250 W/cm^2^ and 25 W/cm^2^ (**b**). Negative shift of the PL band under excitation at 442 nm (**c**).

Rhodamine molecules are known to be degraded under intense light exposure and repeated excitation leading to a decrease of the PL efficiency [25]. The fluorescence response of the sol-gel layers exposed to γ photons will be obtained using two wavelengths: 442 nm and 488 nm. It is thus essential to find the best conditions, in terms of power density and acquisition time, in order to avoid a potential photobleaching effect due to visible exposure while probing the γ exposed R6G TiO_2_-SiO_2_ sol-gel layers. To this aim, we have studied the photodegradation using these two particular wavelengths, by varying both power densities (filters at 1%, 0.1% and 0.01%) and exposure times up to 10 min. The results regarding the photodegradation under a 442 nm excitation are presented in Figure 5a at 5.8 kW/cm^2^ (black), 580 W/cm^2^ (red) and 58 W/cm^2^ (blue). For the highest power density, we can observe a very strong decrease in the PL efficiency down to roughly 33% of the initial PL after 10 s of irradiation while a stabilization seems occuring for higher exposure times. As expected, R6G is less affected when the power density is decreased. As for instance, using the lowest one possible in this experiment (58 W/cm^2^), 99% of the initial PL intensity is still present after a probing duration of 10 s. The exact same experiment was performed for the 488 nm excitation and the results are depicted in Figure 5b. For this latter, we can observe the same behavior insofar as a dramatic decrease of the PL occurs for high power densities. As a consequence, in order to probe the layers exposed to γ-rays and avoid any photobleaching effect, all the samples presented in the following part are characterized at 58 W/cm^2^ (442 nm) and 25 W/cm^2^ (488 nm) with an acquisition time of 1 s. Under these specific conditions, the probing process does not alter R6G molecules incorporated in the sol-gel matrix.

Additionally to the passivation of R6G, we can observe a negative shift of the PL band as illustrated in Figure 5c under excitation at 442 nm. As suggested by Figure 5a, the negative shift is more and more important by increasing the power density to reach a blue shift of 6.4 nm and 2.6 nm after a 600 s irradiation time for the highest (5.8 kW/cm^2^) and lowest power densities (58 W/cm^2^), respectively. The same behavior is obtained by exciting at 488 nm. Interestingly, such blue-shifting was also observed by decreasing the R6G concentration [27]. In our particular case, we can reasonably presume that during the intense light exposure, R6G is degraded by a series of bond breaking events, destroying chromophoric groups and explaining the negative shift observed in Figure 5c.

### 3.3. PL Properties after γ-ray Exposure

**Figure 6:**
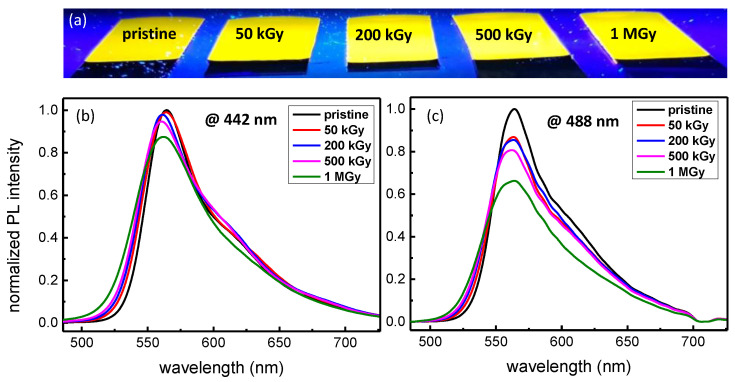
PL study of TiO_2_-SiO_2_ sol-gel doped with R6G exposed to γ-rays with accumulated doses of 50 kGy, 200 kGy, 500 kGy and 1 MGy. Visual observation under a 365 nm (8 W) excitation (**a**). PL spectra under excitation at 442 nm (**b**). PL spectra under excitation at 488 nm (**c**). The power densities are 58 W/cm^2^ and 25 W/cm^2^ for the 442 nm and 488 nm excitation while the exposure time is 1 s. It is to note that the PL was normalized with respect to the maximum PL intensity obtained for the pristine sample.

After the sol-gel film preparation, as described in Section 2, samples were submitted to γ-rays at room temperature and at different accumulated doses up to 1 MGy. More specifically, the effect on their PL properties was investigated and all the results are presented in Figure 6. First of all, we have made a visual observation under excitation at 365 nm (Figure 6a). In the present image, all the accumulated doses are represented (50 kGy, 200 kGy, 500 kGy and 1 MGy) while one pristine layer was only exposed to UV photons. It is evident that TiO_2_-SiO_2_ sol-gel films successfully withstand the hard γ-rays exposure even for the highest accumulated dose (1 MGy). In addition, a quantitative study was performed by recording the PL spectra under excitation at 442 nm (Figure 6b) and 488 nm (Figure 6c). The sol-gel layers were probed by using the parameters (power density and exposure time) defined in Section 3.2, so that potential changes in the PL intensity are only caused by the γ-rays. More specifically, the PL under excitation at 442 nm remains largely satisfying, where 90% of the initial PL intensity can be observed despite the 1 MGy accumulated dose. These results are confirmed by probing the samples at 488 nm: the PL signal is still intense, whatever the accumulated dose, to reach roughly 65% of the pristine PL intensity. Additionally to the PL investigation, the layers were probed by microscopic observations revealing that no damage or crack are induced whatever the accumulated ionizing dose.

### 3.4. FTIR Spectroscopy

**Figure 7:**
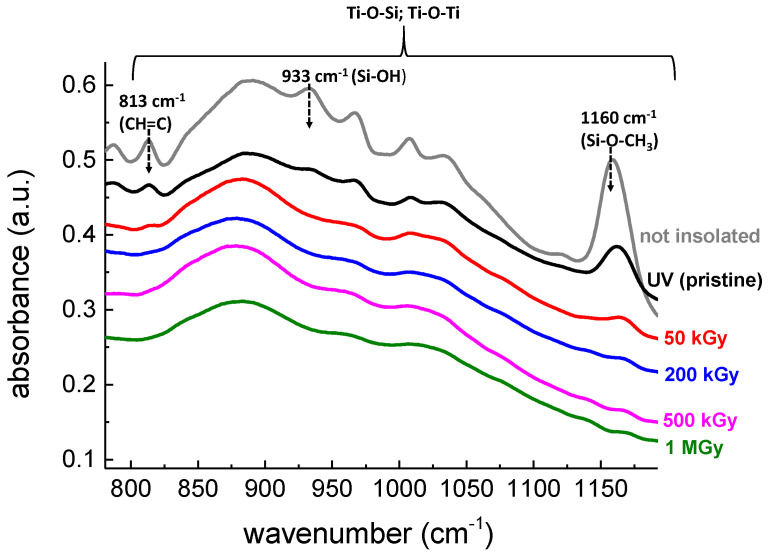
FTIR spectroscopy of organo TiO_2_-SiO_2_ films doped with R6G deposited on Si substrate. The graph represents the non-exposed film (grey line), the UV-exposed film (black line) and the samples exposed to γ-rays with different accumulated doses of: 50 kGy (red line), 200 kGy (blue line), 500 kGy (magenta line) and 1 MGy (green line). All the layers exposed to γ-rays were first exposed to UV photons. For better visualization, spectra are vertically shifted.

FTIR spectroscopy is a powerful technique allowing the chemical and structural characterization of sol-gel materials [30]. It is to remind that the TiO_2_-SiO_2_ sol-gel films doped with R6G are photosensitive with respect to UV photons inducing a photopolymerization process. As observed by others authors, it is possible to monitor the polymerization degree of hybrid TiO_2_-SiO_2_ layers with respect to light exposure [16,29] by investigating the [800 cm^−1^–1250 cm^−1^] range, where inorganic and organic network modifications can be observed. In order to understand the evolution of R6G doped sol-gel layers under γ-rays exposure, FTIR spectra were recorded in this particular region for all the accumulated doses and synthetized in Figure 7. This investigated range is assigned to Ti-O-Ti, Ti-O-Si optical responses and their potential combinations [16] through a large band peaking at roughly 890 cm^−1^. Interestingly, three narrower bands are present and are of great interest regarding the polymerization evolution. These bands peaking at 813 cm^−1^, 933 cm^−1^ and 1160 cm^−1^ correspond to the stretching vibration of CH=C, Si-OH and Si-O-CH_3_ groups, respectively [26,29]. To clearly understand the process involved during the polymerization, a non-exposed film (grey line), only submitted to thermal treatments, is presented where the three bands of interest remain intense. After being exposed to UV photons (365 nm, 180 J/cm^2^), the corresponding FTIR spectrum (black line) reveals a decrease in the 813 cm^−1^ band intensity compared with the unexposed films, thus evidencing bond breakings of CH=C and initiation of a polymerization process. Additionally, a decomposition of Si-OH and Si-O-CH_3_ groups can be observed through a decrease of their respective intensities leading to the creation of Ti-O-Si, Ti-O-Ti or Si-O-Si bonds. The layers exposed to UV photons were also exposed to γ-rays with different accumulated doses of 50 kGy (red line), 200 kGy (blue line), 500 kGy (magenta line) and 1 MGy (green line). Interestingly, despite a first UV insolation, the samples exposed to γ photons are still photosensitive. A further polymerization process is evidenced through a strong decrease in the 813 cm^−1^, 933 cm^−1^ and 1160 cm^−1^ bands. Obviously, the decrease is more pronounced for higher accumulated doses where it seems that the consumption of the organic network is total. Finally, after a 1 MGy accumulated dose, only the large band peaking at 890 cm^−1^ is present clearly revealing that Ti-O or Si-O bonds are unaffected and successfully withstand the irradiation.

However, despite the incorporation of R6G, we have no noticeable signature of the organic dye in the FTIR spectra. This behavior was already observed in [23] where typical bands attributable to R6G were not detected once incorporated in a silica matrix. Moreover the R6G being present in a trace quantity in the samples, due to the low thickness of the thin layers and therefore the low amount of material, was more difficult to detect by FTIR. It is to remind that Figure 7 displays the results regarding TiO_2_-SiO_2_ sol-gel films doped with R6G. Undoped sol-gel films were also evaluated using FTIR spectroscopy (not shown here) and the same behavior as Figure 7 occurs.

### 3.5. Radiation Induced Refractive Index Change

**Figure 8:**
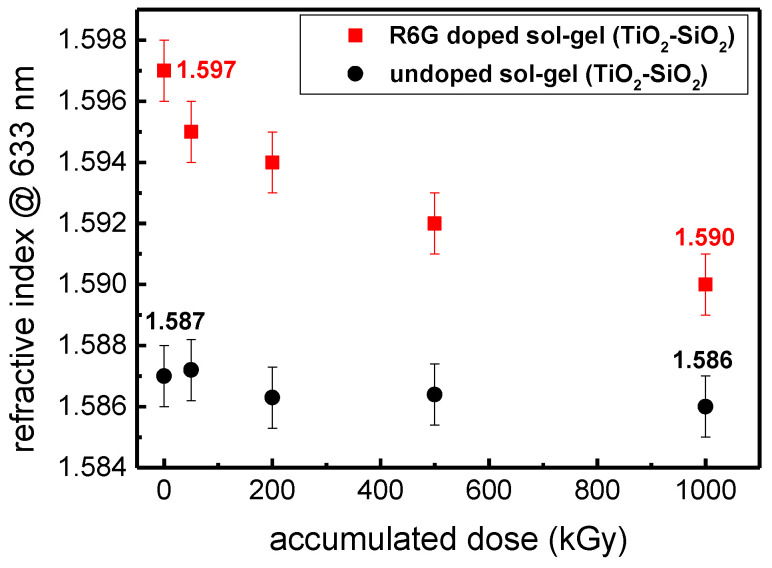
Refractive index (633 nm) evolution for different accumulated doses of: 0 kGy (pristine sample), 50 kGy, 200 kGy, 500 kGy and 1 MGy. The investigated films are a R6G doped sol-gel layer (red square) and an undoped TiO_2_-SiO_2_ film (black line). The films were deposited on a borofloat 33 substrate.

In addition to PL and FTIR studies, the refractive index was also investigated using the M-lines spectroscopy. This value is of great interest regarding the development of photonic sensors where a strong refractive index allows having the best performances. First, we have measured the refractive index of a R6G doped sol-gel and the results are represented in Figure 8 (red square). A refractive index of 1.597 is measured at 633 nm for the not-irradiated sample with γ-rays. A decrease of the refractive index can be observed after the sample has been irradiated. Obviously, the degradation is more important by increasing the accumulated dose. As for instance, a decrease of the refractive index in the range of 7 × 10^−3^ can be achieved at 1MGy. However, in order to evaluate if the degradation is caused by the organic dye contribution or induced by the precursors used during the sol-gel elaboration, a second TiO_2_-SiO_2_ sol-gel film was prepared without R6G, exposed to γ-rays and characterized using the same approach (black line). The primary observation is that the refractive indices are globally lower compared with the R6G doped sol-gel with an initial value of 1.587 before exposure to γ photons. However, the refractive indices seem to be rather stable and unaffected despite the different accumulated doses. This result clearly evidences that the refractive index change observed for the R6G doped films exposed to different accumulated doses is due to the R6G contribution.

## 4. Conclusions

In the present paper, we have investigated the effect of hard γ-ray photons (1.17 MeV and 1.33 MeV) on the properties of an organic-inorganic TiO_2_-SiO_2_ thin films doped with R6G. After incorporation in the host matrix, the layers exhibit a strong absorption band peaking at 526 nm while a very intense PL signal is present at 564 nm. The layers, once exposed to UV photons (365 nm–180 J/cm^2^), were submitted to different accumulated γ-doses up to 1 MGy. PL signals are still largely intense to reach roughly 90% and 65% of the initial PL intensity under excitation at 442 nm and 488 nm, respectively for the highest accumulated dose. In addition to the PL study, the refractive index change was monitored through M-lines spectroscopy. This latter reveals that a decrease in the refractive index can be observed with a maximum change of 7 × 10^−3^ after a 1 MGy accumulated dose regarding the doped sol-gel while no significant variation is highlighted in the undoped sol-gel (host matrix). Finally, R6G doped sol-gel layers were characterized by FTIR spectroscopy. Even though the films were preliminary exposed to UV photons, a further polymerization still occurs under γ-rays exposure up to 1 MGy and is related to the consumption of CH=C, Si-OH, and Si-O-CH_3_ groups while the band related to Ti-O or Si-O is unaffected. All these promising results show that both TiO_2_-SiO_2_ sol-gel films and R6G molecules withstand the hard γ-ray exposure, can operate in a variety of radiation-rich environments and could resist to the X-ray inspection phases in aerospace domain.

## Data Availability

Not applicable.
